# Musicogenic seizures in temporal lobe epilepsy: Case reports based on ictal source localization analysis

**DOI:** 10.3389/fneur.2023.1072075

**Published:** 2023-02-20

**Authors:** Ionut-Flavius Bratu, Adriana Elena Nica, Irina Oane, Andrei Daneasa, Sergiu Stoica, Andrei Barborica, Ioana Mindruta

**Affiliations:** ^1^Epilepsy Monitoring Unit, Neurology Department, University Emergency Hospital Bucharest, Bucharest, Romania; ^2^Intensive Care Unit, University Emergency Hospital Bucharest, Bucharest, Romania; ^3^Neurosurgery Department, Brain Institute, Monza Hospital, Bucharest, Romania; ^4^Physics Department, University of Bucharest, Bucharest, Romania; ^5^Faculty of Medicine, Carol Davila University of Medicine and Pharmacy, Bucharest, Romania

**Keywords:** musicogenic epilepsy, temporal lobe, focal cortical dysplasia, anti-GAD, independent component analysis, ictal source localization

## Abstract

Musicogenic epilepsy is a rare form of reflex epilepsy in which seizures are provoked by music. Different musicogenic stimuli have been identified: pleasant/unpleasant music or specific musical patterns. Several etiologies have been uncovered, such as focal cortical dysplasia, autoimmune encephalitis, tumors, or unspecific gliosis. In this article, we report two patients with musicogenic seizures. The first patient was diagnosed with structural temporal lobe epilepsy. Her seizures were elicited by music that she liked. Interictal and ictal video-electroencephalography (video-EEG) and signal analysis using independent component analysis revealed the right temporal lobe seizure onset extending over the neocortical regions. The patient underwent right temporal lobectomy (including the amygdala, the head, and the body of the hippocampus) and faced an Engel IA outcome 3 years post-surgery. The second patient was diagnosed with autoimmune temporal lobe epilepsy (GAD-65 antibodies). Her seizures were triggered by contemporary hit radio songs without any personal emotional significance. Interictal and ictal video-electroencephalography (video-EEG) and independent component analysis highlighted the left temporal lobe seizure onset extending over the neocortical regions. Intravenous immunoglobulin therapy was initiated, and the patient became seizure-free at 1 year. In conclusion, musicogenic seizures may be elicited by various auditory stimuli, the presence or absence of an emotional component offering an additional clue for the underlying network pathophysiology. Furthermore, in such cases, the use of independent component analysis of the scalp EEG signals proves useful in revealing the location of the seizure generator, and our findings point toward the temporal lobe, both mesial and neocortical regions.

## Introduction

Musicogenic epilepsy is a rare form of reflex epilepsy (1), with an estimated prevalence in the general population of 1 in 10 million people (2), in which seizures are provoked by music. Various etiologies have been reported such as focal cortical dysplasia (FCD) type I (3), astrocytoma (4), glial scars (5), demyelinating lesions (6), and autoimmune encephalitis (7). However, in most cases, a structural lesion could not be ascertained (5). The treatment of musicogenic seizures usually ranges from the avoidance of the musical triggers together with anti-seizure medication to epilepsy surgery (2). As there is emerging evidence of an association between musicogenic seizures and anti-glutamic acid decarboxylase antibodies (anti-GAD abs) encephalitis, the focus progressively shifts toward immunotherapy (8, 9).

This article aimed to present two cases of musicogenic epilepsy of different etiologies (FCD type IIA and anti-GAD abs encephalitis) that involve the left or right temporal lobe, with their particularities of diagnosis and management of subsequent learning points.

## Case 1

### Case description

A 30-year-old right-handed woman with a medical history of type 1 diabetes mellitus (DM), distal sensory diabetic polyneuropathy, Stargardt disease, nephrolithiasis, and anxiety-depressive disorder presented to our neurology department for recurrent epileptic seizures. She described her habitual diurnal episode as an ascending epigastric sensation (accounted for as fear), followed by complex visual hallucinations, nausea, and verbal automatisms. During the focal seizures that lasted up to 1 min, the patient maintained contact and exhibited facial cyanosis. She had no postictal deficit. The patient emphasized that listening to music triggered her diurnal seizures, particularly music that she liked. She had her first seizure at the age of 29 years. She was the result of an uneventful pregnancy and had normal psychomotor development. The patient had no history of cranial-cerebral trauma, febrile convulsions, or neuro-infections. The neuropsychological examination was normal, but she had an anxiety-depressive disorder. As to her familial medical history, the only disorders mentioned were type 1 DM in one of her children and Stargardt disease in two of her children and her sister. At the moment of her presentation to our clinic, she was on levetiracetam (500 mg bd) and lamotrigine (100 mg bd), but she was still experiencing daily seizures. Her longest seizure-free period was 2 months. Her general and neurological clinical examinations revealed severely reduced visual acuity in both eyes, particularly in the macular field.

### Diagnostic assessment

**Figure 1:**
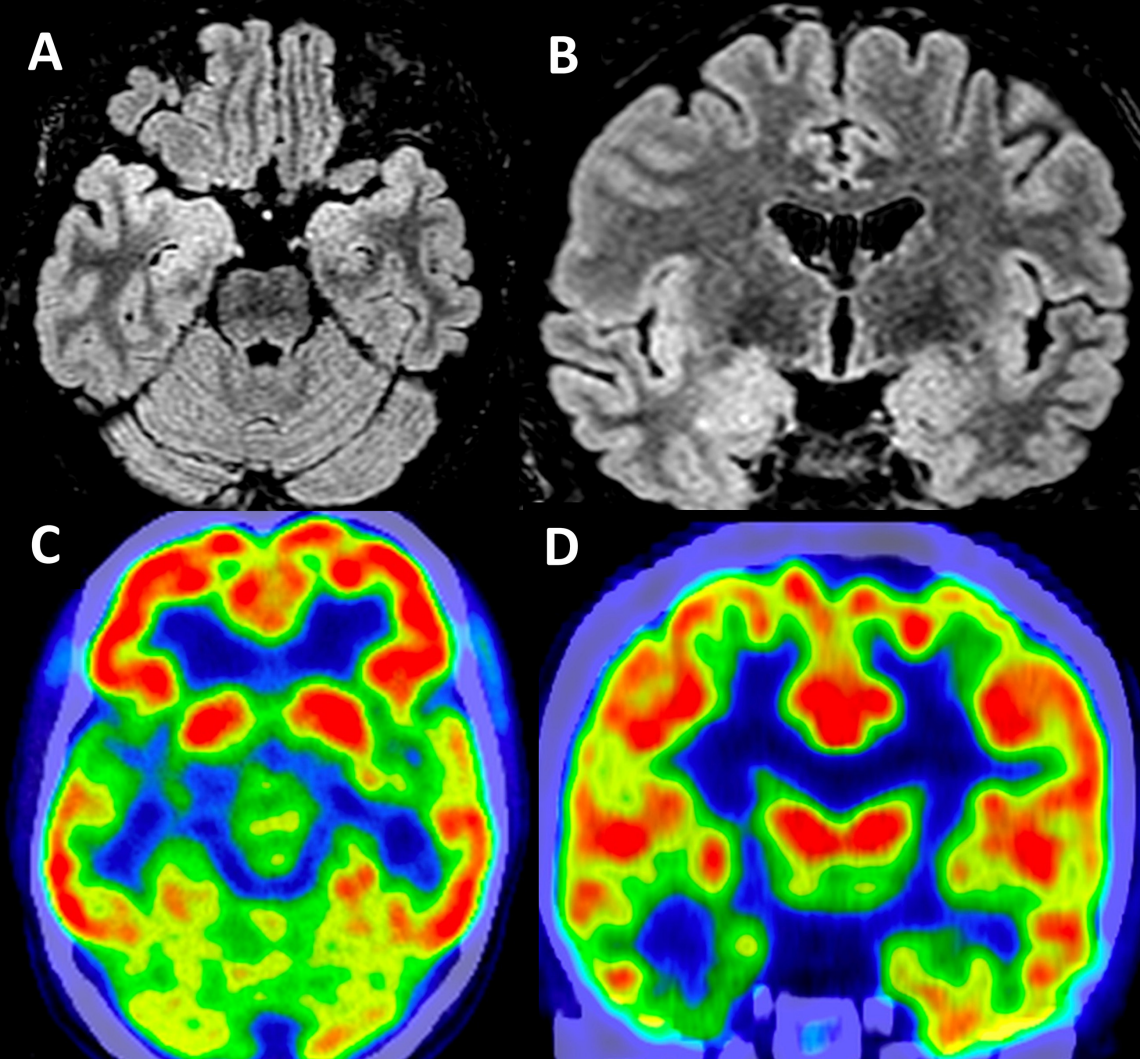
Structural and functional brain imaging in Case 1. Interictal cerebral MRI scan—axial **(A)** and coronal **(B)** sections—showing right amygdala and hippocampus FLAIR hyperintensities. Interictal functional imaging using 18FDG-PET—axial **(C)** and coronal **(D)** sections—showing extensive right temporal lobe hypometabolism: the temporal pole, mesial and basal areas, and superior temporal and Heschl gyri.

**Figure 2:**
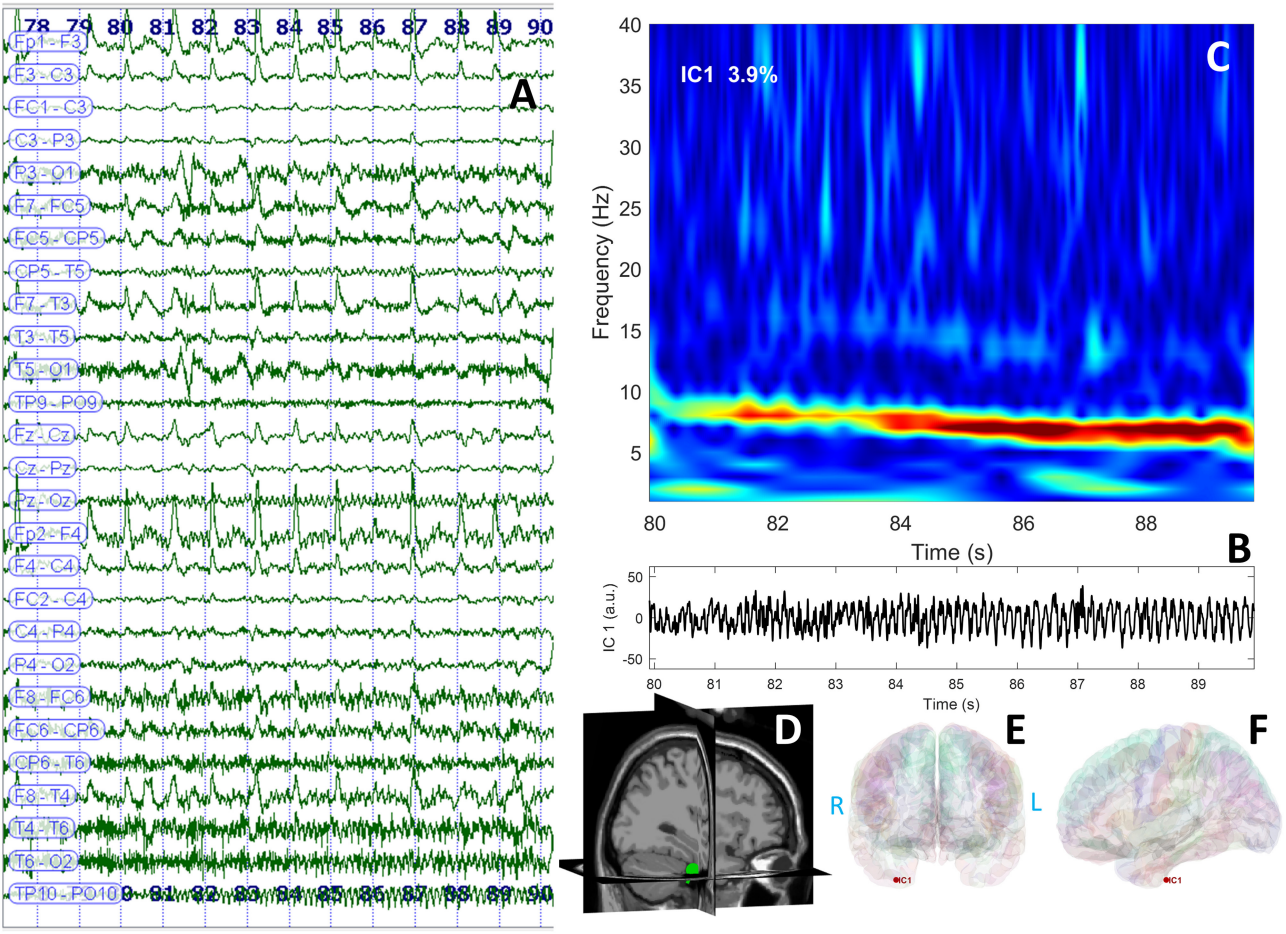
EEG data displaying rhythmic ictal discharge over the right temporal leads spreading toward the suprasylvian and contralateral regions after the clinical onset **(A)**; independent component analysis identified a theta discharge represented in the time domain **(B)** and frequency domain **(C)**; the dipole was represented in the right temporal region of the patient's cerebral MRI (3D view) **(D)**; and cortical surface reconstruction—coronal **(E)** and sagittal **(F)** sections.

Long-term video-EEG monitoring showed interictal epileptiform discharges during wakefulness and NREM sleep, predominantly in the right frontal-temporal leads (Fp2–F8, F8–T4, T4–T6, and TP10–PO10). Two reflex seizures were recorded within 15 s after listening to one song that she liked. Clinically, she alerted the medical personnel that she felt nauseous, and she touched her epigastric region with her left hand. During the first few seconds, she could answer and follow commands, but after that, she lost awareness and exhibited left upper limb and oral-alimentary automatisms. She also presented a postictal cough. Electrically, the seizures started in the right frontal-temporal leads (F8, T4, and FC6). The interictal cerebral magnetic resonance imaging (MRI) scan showed right amygdala, hippocampus, and basal temporal lobe T2-weighted/FLAIR hyperintensities. The interictal cerebral FDG-PET scan revealed extensive right temporal lobe hypometabolism: temporal pole, mesial and basal areas, and superior temporal and Heschl gyri (Figure 1). To better characterize the electrical seizure onset and the early propagation network, we performed independent component analysis (ICA) of scalp EEG signals (Figure 2A) (10, 11) using the infomax algorithm (12) as implemented in EEGLAB (13). One independent component (IC) was visually selected by an expert epileptologist based on the IC activation pattern (Figure 2B) and its time–frequency decomposition (Figure 2C). Afterward, an equivalent current dipole (ECD) model was fitted to the component (Figures 2D–F). In this case, the dipole was located in the right anterior temporal basal region (Figures 2D–F). Due to patient's history and paraclinical investigations, a structural etiology was presumed, and the patient underwent extended right temporal lobectomy (including the mesial temporal lobe structures and the lateral neocortex—superior, middle, and inferior temporal gyri, as well as the temporal-basal cortex). The histopathological examination revealed FCD type IIA. The follow-up cerebral MRI scan and video-EEG recording at 6 months showed no structural abnormalities or epileptiform activity. The patient was Engel IA outcome at 3 years post-surgery.

## Case 2

### Case description

A 40-year-old right-handed woman with a medical history of type 1 DM with implanted glycemia sensor presented to our neurology department for recurrent seizures. She had a mean of three focal aware seizures per day during wakefulness. She described her habitual diurnal episode as a feeling that time expands (if she had a conversation, the words would expand indefinitely), then she would not understand what is said to her, she could not speak, but she could continue the activity that she was engaged in. During the seizures that lasted up to 1 min, the patient maintained eye contact and postictally she had anomia for up to 30 min. She emphasized that listening to music triggered her seizures, particularly listening to a specific musical rhythm or a peculiar pattern that has been linked to a contemporary hit radio song. She could not attribute a personal emotional aspect to the ictogenic music. She had her first seizure at the age of 36 years. She was the result of an uneventful pregnancy and had normal psychomotor development. The patient had no cranial-cerebral trauma, no febrile convulsions, or neuro-infections in the past, and her familial medical history did not show relevant afflictions. The neuropsychological examination was normal. At the moment of her presentation to our clinic, she was not taking any anti-seizure medication even though she had been prescribed levetiracetam (500 mg bd). Her longest seizure-free period had been 6 months (the longest period during which she could avoid musicogenic triggers). Her general and neurological clinical examinations were normal.

### Diagnostic assessment

**Figure 3:**
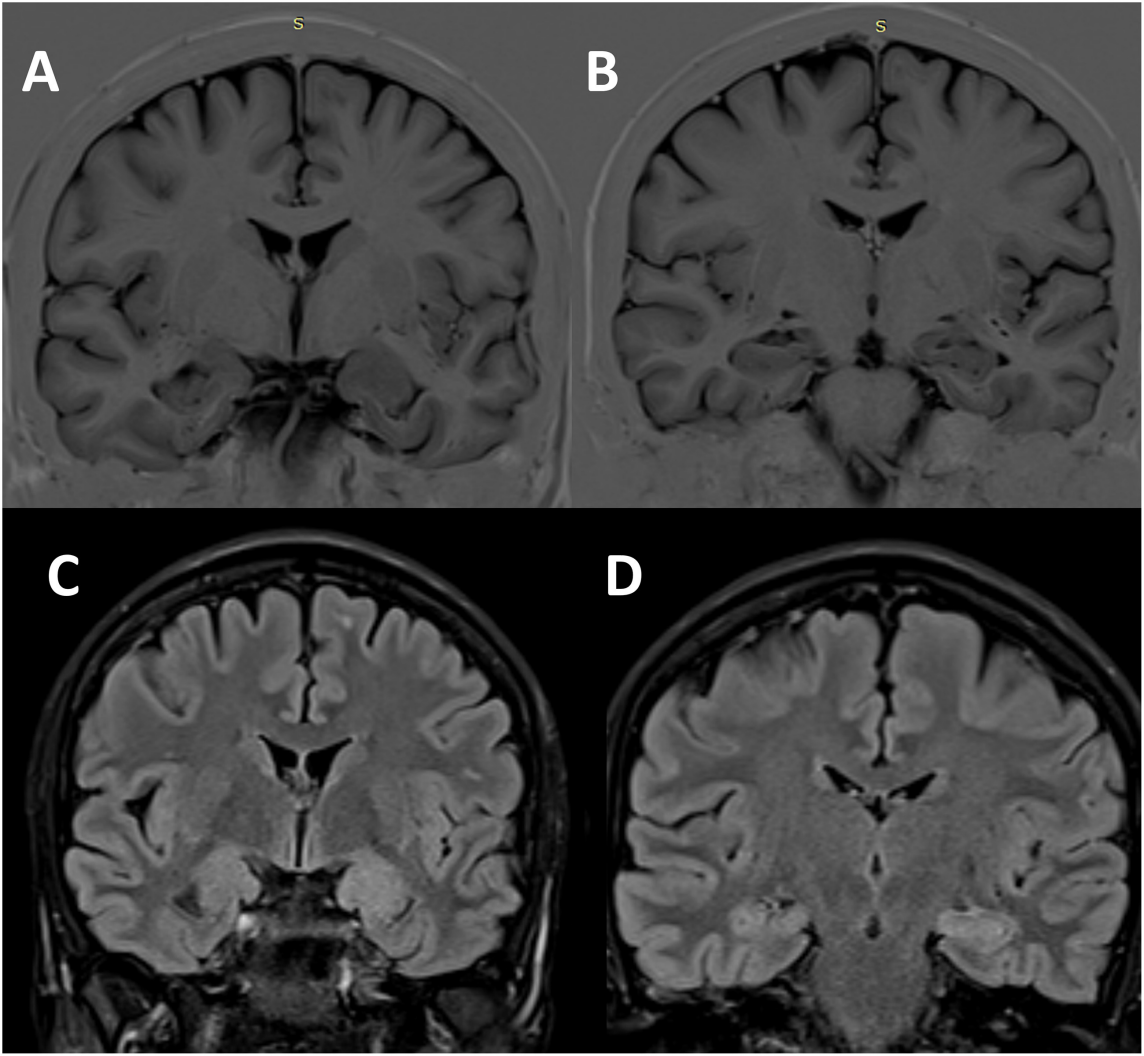
Structural brain imaging in Case 2. Interictal cerebral MRI scan—coronal sections—showing the enlargement of the left amygdala **(A)** and hippocampus **(B)** on T1-inversion recovery pulse sequence, as well as FLAIR hyperintensities in the same regions [**(C)** amygdala; **(D)** hippocampus].

**Figure 4:**
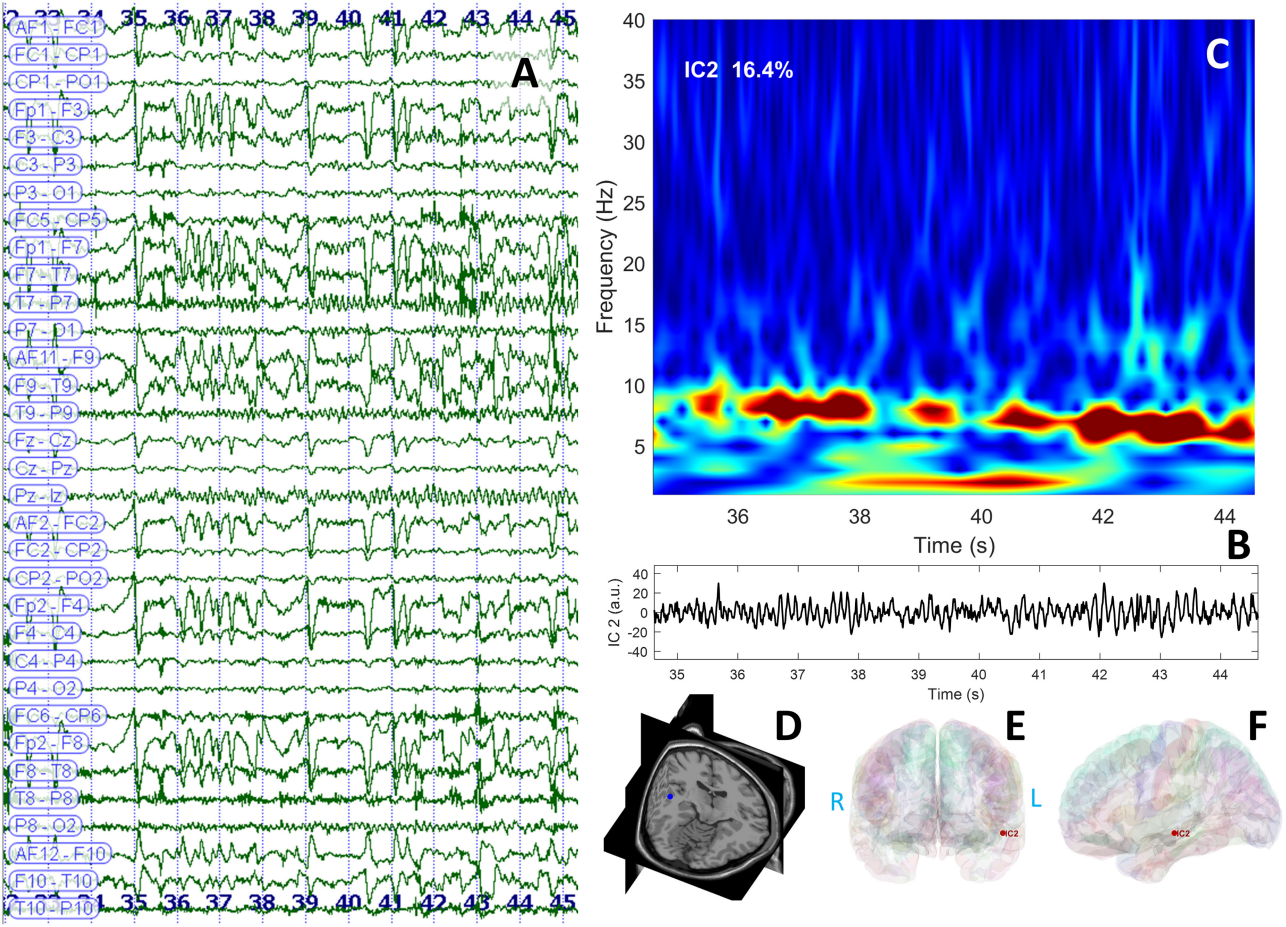
EEG data displaying rhythmic ictal discharge over the left temporal leads spreading toward the suprasylvian and contralateral regions after the clinical onset **(A)**; independent component analysis identified a theta discharge represented in the time domain **(B)** and the frequency domain **(C)**; the dipole was localized in the left temporal neocortical region of patient's cerebral MRI (3D view) **(D)**; and cortical surface reconstruction—coronal **(E)** and sagittal **(F)** sections.

The interictal brain MRI scan revealed an increase in the volume of the left amygdala and the hippocampus and FLAIR hyperintensities in the same regions (Figure 3). Long-term video-EEG monitoring showed interictal epileptiform discharges during wakefulness and NREM sleep predominantly over the left temporal leads (Fp1–F7, F7–T7, T7–P7, and F9–T9). Two seizures were recorded after the patient was exposed to the habitual auditory stimuli. She experienced her seizures in 15 min after being exposed to random contemporary hit radio songs and in 40 s after being exposed to one song that she believed to be ictogenic. Clinically, she alerted the medical personnel when the seizure started (she reported afterward the sensation of time expansion), and then she exhibited speech arrest and also right upper limb and oral-alimentary automatisms. Postictally, she had anomic elements and paraphasia. Electrically, the seizures started in the left frontal-temporal leads with theta discharge over the temporoparietal region at the clinical onset (Figure 4A). The independent component analysis as described in the case before [with the exception that the SOBI (14) algorithm worked better than infomax for this patient] highlighted one component encoding a sustained ictal theta discharge (Figures 4B, C), with dipoles localized in the left superior temporal gyrus (Figures 4D–F). Due to the association between DM type 1 and seizures, the patient underwent autoimmune testing. The bloodwork showed an elevated titer of anti-GAD abs (2,000,000 UI/ml). In this context, the patient was diagnosed with autoimmune encephalitis with anti-GAD antibodies and was started on intravenous immunoglobulins (IVIG) (2 g/kg). She did not exhibit any side effects from the immunotherapy. Following the IVIG pulse therapy, we tried to induce another musicogenic event, but even after prolonged exposure to the ictogenic music, no seizure could be elicited either clinically or electrically. The follow-up MRI scan at 3 months showed a significant reduction in the hyperintensities and the volume of the left amygdala and the hippocampus. Until the moment of this report, the patient had received only one pulse of IVIG. However, she is still on anti-seizure medication, and she can now successfully avoid auditory triggers by wearing headsets. Her condition is stable, and she did not develop any further neurological deficit or cognitive impairment.

## Discussion

The main differences between musicogenic and elementary stimulus audiogenic seizures involve the characteristics of epilepsy and the complexity of auditory triggers (15). In the case of musicogenic epilepsy, seizures typically start later in life, the average onset age being 28 years (16) as opposed to audiogenic epilepsy with a more commonly younger onset (2). Musicogenic seizures are triggered by the melodic or harmonic combinations of sounds with complex features [e.g., timbre and bandwith (17, 18)] that represent specific triggers for each individual. Audiogenic seizures are usually triggered by a sudden non-specific sound (15). On the spectrum of musicogenic to audiogenic epilepsy, in investigating the specificity of the stimuli, Weiser et al. (16) highlighted in their review that music was the only provoking stimulus in 78% of the patients, with less specific combinations of sounds and music in 14% of the patients. In only 4% of individuals, sounds were identified to be the only ictogenic triggers (16). Musicogenic seizures typically occur with longer latency, even after several minutes of stimulation (19, 20), as opposed to audiogenic seizures that occur within a short time frame after the sound. Garcia-Casares et al. (21) observed that musicogenic triggers included music with emotional significance to the patient, which may be any kind of music, a specific genre of music or song and music with a certain wave frequency or rhythm. The musical complexity of the ictogenic stimuli and the longer latency indicate a cognitive or emotional aspect of the musicogenic trigger, which differs from the more primary response when random sounds or noise are a trigger (5). However, the longer latency in the cases of musicogenic-elicited seizures was not always associated with emotional feeling buildup (22). Both our patients had seizures when exposed to musicogenic triggers after a latency period of 15–40 s, but only in the right temporal lobe epilepsy case, the music involved an emotional component (seizures to music that she liked, the most probable to elicit an epileptic fit being her favorite music). Salimpoor et al. (23) demonstrated increased functional connectivity between cortico-striatal areas (superior temporal gyrus–auditory cortex, inferior frontal gryus, and nucleus accumbens) in parallel with the pleasure perceived (“reward value”) during music listening. Moreover, Nuara et al. (24) suggested that in the context of patients who emphasize an emotional component of the music triggering their seizures, particularly patients with the right-hemisphere epileptogenic zone, the “emotional glow” of perceived music is a possible independent trigger in musicogenic epilepsy, allegedly involving extra-auditory regions (25). This could explain the reflex seizures described in Patient 1 who only experienced them when listening to music that she liked.

Although the musicogenic trigger is specific for the affected individual, triggers vary broadly across individuals with musicogenic epilepsy. In terms of audiogenic or musicogenic triggers, they can vary from the sound of a vacuum cleaner (26) or a sequence of simple tones (21, 27) to the voices of particular singers [a throaty “metallic” singer's voice (18)] and instrumental music (16). In both presented cases, the patients had seizures when exposed to vocal music, not purely instrumental.

The triggering music can be actively heard (listened to) as background music, imagined, or dreamed, and in some cases, the seizures are elicited only when the specific piece of music was actively performed (hearing the piece or silently mimicking the performance of the piece was not a trigger) (17, 26–30). Both our patients exhibited seizures only when listening to music.

For most individuals with musicogenic epilepsy, the trigger is a specific piece or type of music. In our first case, the patient exhibited seizures when exposed to a specific genre of music that she liked. Our second patient presented seizures when listening to contemporary hit radio songs.

In 14 cases (17%) of a series of 83 patients, seizures were only present when triggered by music. These 17% were consistently seizure-free when not exposed to music (16). If our first patient had both spontaneous seizures and musicogenic reflex ones, our second patient exhibited solely musicogenic reflex seizures.

In the study of Avanzini et al. (22) there was a melodic and rhythmic stimuli predominance as opposed to purely melodic, rhythmic, or textual importance. Furthermore, the familiarity or affective content accounted for almost 30% of all musicogenic-induced seizures, and much more than that, the novelty, particularity of the genre, part of the song, or its purely instrumental form (1–20%). Among the instruments, the piano and organ were the most effective ones in generating seizures. However, the level of musical training pointed out that patients who had no specific interest in music or were amateurs had the highest chance of developing musicogenic seizures (22). Both our patients had no musical training or specific interest in the domain.

Wieser et al. (16) indicated in their work that patients with musicogenic epilepsy exhibited right lateralization in 61% of the cases and temporal lobe epilepsy in 75%. Furthermore, different reports incriminated, using scalp EEG and/or SPECT, the right temporal lobe (31–33) or the left temporal one (34). Intracranial EEG studies incriminated both temporal lobes (the right more frequent than the left) and the seizure onset zone involving both mesial and neocortical structures (15, 35–40). We presented one case of left temporal lobe epilepsy and one of right temporal lobe epilepsy.

The variability of whether the musicogenic seizures were elicited by music associated with emotional significance was hypothesized to be superposable over the network involved in seizure generation: neocortical hubs (superior temporal gyrus and Heschl gyri) or mesial temporal hubs (amygdala and hippocampus) (16, 22, 38, 41–43). Diekmann et al. (25) revealed that using fMRI in patients in whom emotions are associated with the music generating the seizures, the musical characteristics *per se* did not appear to be the crucial elements leading to the epileptic seizures. BOLD signal changes were seen by Diekmann et al. (25) in areas supposedly related to cognitive processing and the regulation of emotions (the left amygdala, the left hippocampal areas, the left dorsomedial prefrontal cortex, the Broca region, the right-sided temporoparietal junction area, the right premotor cortex, and the cerebellum—especially the VIth, VIIth, and VIIIth lobules) (44–49). Another neural hub that is involved in the emotional aspects of music processing (50–52) and is highlighted by means of fMRI (53) to be recruited during musicogenic seizures is the orbitofrontal cortex.

Anti-GAD abs have been found in patients with limbic encephalitis and pharmacoresistant focal temporal epilepsy (54). Vianello et al. (55) revealed an increase in the spontaneous activity of hippocampal neurons in culture caused by the suppression of inhibitory potentials mediated *via* anti-GAD antibodies. They suggested interference with the GABA function and consequently with neuronal inhibition, thus supporting a pathogenetic role of anti-GAD abs in the development of epilepsy. Furthermore, Stagg et al. (56) emphasized that in patients with epilepsy, a high anti-GAD abs titer is associated with low cortical GABA levels.

The involvement of the temporal lobes in epilepsy with anti-GAD-abs can be asymmetrical, with patients achieving seizure freedom after unilateral temporal lobe resection (57, 58). However, clinical, EEG, and FDG-PET findings suggest a widespread disease not restricted to the temporal lobe (mesial temporal lobe sclerosis) but also involving the insular cortex (59). Anti-GAD-ab-mediated epilepsy is often pharmacoresistant. It is moderately responsive to immunotherapy (steroids, intravenous immunoglobulin, or plasma exchange), and more aggressive immunosuppressants such as rituximab and/or cyclophosphamide are often necessary (60). Several articles (61, 62) suggested musicogenic reflex seizures to be a distinctive seizure type in patients with epilepsy with anti-GAD-abs. The association between type 1 DM, high anti-GAD-abs titer, and right temporal lobe musicogenic seizures has been described before (63). Smith et al. (9) found in their study, a serological association of musicogenic epilepsy with high anti-GAD65 IgG titers. All their patients had temporal lobe epilepsy, with right temporal lobe epilepsy being more common among patients with musicogenic epilepsy than in patients with non-musicogenic GAD65 epilepsies.

Previous articles have demonstrated that the network of musicogenic seizures involves the limbic system and the auditory temporal neocortex (64). Taking this into consideration, in the first case, the patient underwent a tailored temporal lobectomy extending posteriorly and superiorly to include the superior temporal gyrus.

One of the limitations is that in the case of the second patient, we did not perform a lumbar puncture with subsequent antibody panel analysis of the cerebral spinal fluid (CSF). In her case, the serum antibody titer was 2,000,000 UI/ml, and we believe that CSF analysis would not have brought additional information that would alter subsequent management and outcome.

### Patient perspective

Both patients are satisfied with the treatment outcome and have returned to their normal lives.

## Conclusion

Reflex musicogenic epilepsy is a rare entity that needs patient-specific management as it has a wide range of etiologies. Moreover, musicogenic triggers may vary and are complex, thus making their identification and management more challenging. The presence or absence of an individual emotional aspect of the ictogenic stimuli offers an additional clue for the underlying network pathophysiology. Furthermore, in such cases, the use of independent component analysis of the scalp EEG signals proves useful in revealing the location of the seizure generator, and our findings point toward the temporal lobe, both mesial and neocortical regions.

## Data availability statement

The raw data supporting the conclusions of this article will be made available by the authors, without undue reservation.

## Ethics statement

Written informed consent was obtained from the individuals for the publication of any potentially identifiable images or data included in this article.

## Author contributions

I-FB and IO: conceptualization, methodology, investigation, data curation, writing—original draft, and writing—review and editing. AN: conceptualization, methodology, investigation, data curation, writing—original draft, writing—review and editing, and funding acquisition. AD and SS: data curation and writing—original draft. AB: conceptualization, methodology, software, formal analysis, writing—original draft, writing—review and editing. IM: conceptualization, methodology, investigation, data curation, writing—original draft, writing—review and editing, and supervision. All authors contributed to the article and approved the submitted version.
